# Hemochromatosis As an Unusual Cause of Pancreatitis in an African-American Female of Child-bearing Age

**DOI:** 10.7759/cureus.7179

**Published:** 2020-03-04

**Authors:** Asif Hitawala, Mohammad Alomari, Shrouq Khazaaleh, Ahmed Alomari, Madhusudhan R Sanaka

**Affiliations:** 1 Internal Medicine, Cleveland Clinic-Fairview Hospital, Cleveland, USA; 2 Internal Medicine, Cleveland Clinic Foundation, Cleveland, USA; 3 Internal Medicine, The Hashemite University, Zarqa, JOR; 4 Gastroenterology and Hepatology, Cleveland Clinic Foundation, Cleveland, USA

**Keywords:** pancreatitis, hemochromatosis, iron overload

## Abstract

Hemochromatosis is a disorder of iron overload whereby there is toxic deposition of iron in various tissues and organs of the body. It can either be hereditary or secondary to some other underlying cause. Patients with mutations in the HFE gene are often predisposed to developing this disorder. It has a wide range of clinical presentation, from non-specific symptoms such as fatigue to overt development of cirrhosis, diabetes and skin pigmentation. We present an unusual case of hemochromatosis where an African-American female of child-bearing age presented to the emergency room with complaints of epigastric pain. She was found to have mildly elevated lipase and liver enzymes. Imaging studies were suggestive of acute-on-chronic pancreatitis with iron deposition in the spleen, pancreas and bone marrow. Her ferritin and transferrin saturation levels were elevated. She was diagnosed with acute-on-chronic pancreatitis secondary to alcoholism and hemochromatosis and treated with phlebotomy with good outcome. This case is one of the few reported cases of hemochromatosis in African-Americans, and emphasizes that even females in child-bearing age group can develop this condition. Elevated ferritin and transferrin saturation levels should prompt evaluation for this disorder.

## Introduction

Hemochromatosis is a well-defined syndrome characterized by toxic accumulation of iron in parenchymal cells of vital organs. It can be caused by mutations in any gene that regulate dietary iron absorption into the body. The iron-driven erythropoiesis is essentially normal [1]. Majority of patients with hemochromatosis are asymptomatic and only a small proportion develop symptoms of iron overload, sometimes with severe manifestations including cirrhosis, diabetes mellitus, cardiomyopathy and joint disease [2]. We describe an unusual case of hemochromatosis leading to pancreatitis.

## Case presentation

A 32-year-old African-American female with past medical history significant for hypertension, pancreatitis, anxiety and left internal carotid artery dissection and thrombus presented to our emergency department with epigastric pain of one-day duration. Her medications included labetalol and lorazepam. She reported drinking two glasses of wine two to three times a week on average, her last drink being a day prior to admission. She had developed acute pancreatitis about a year ago which was attributed to alcoholism. Imaging studies at that time did not show signs of chronic pancreatitis. There was no significant family history of pancreatitis.

On arrival, her heart rate was 101/min and blood pressure was 177/116 mmHg. The remainder of the vital signs were otherwise within normal limits. Physical examination was significant for mild epigastric and left upper quadrant tenderness.

**Figure 1:**
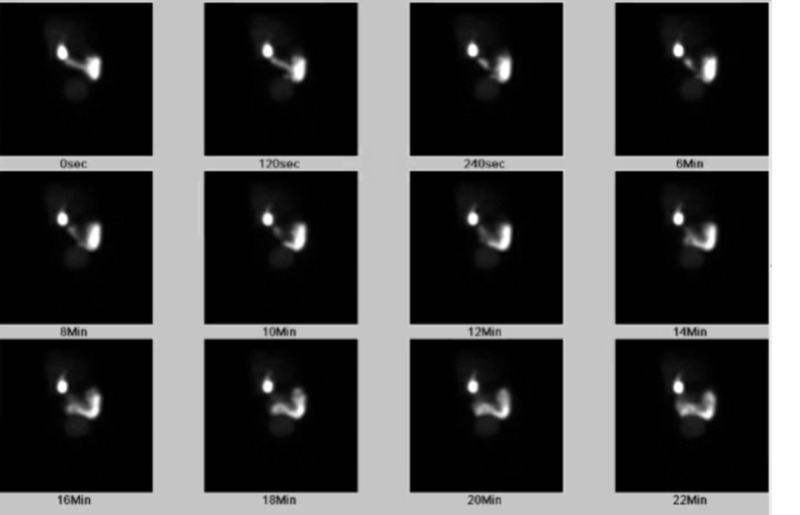
A hepatobiliary iminodiacetic acid scan. The figure shows the gallbladder ejection fraction of 6%.

Initial laboratory work-up was remarkable for hemoglobin 14.2 gm/dL (normal: 11.5-15.5 gm/dL), hematocrit 41.8% (normal: 36-46%), aspartate aminotransferase 197 U/L (normal: 7-40 U/L), alanine aminotransferase 63 U/L (normal: 0-45 U/L), alkaline phosphatase 124 U/L (normal: 34-123 U/L) and total bilirubin 1.5 mg/dL (normal: 0.2-1.3 mg/dL). Lipase was mildly elevated at 69 U/L (normal: 16-61 U/L). Triglycerides were 67 mg/dL (normal: 35-150 mg/dL). Fasting blood glucose levels were within normal range of 70-100 mg/dL. Urine toxicology screen was negative, which included phencyclidine, cocaine, amphetamines and ethanol. Blood alcohol level was not detectable. IgG-4 levels were checked to rule out possible autoimmune pancreatitis and were within normal limits. Computed tomography angiography of the abdomen and pelvis showed mild hepatomegaly and hepatic steatosis but no aortic or major vessel dissection or aneurysmal dilatation. A hepatobiliary iminodiacetic acid scan showed a decreased ejection fraction of 6% (Figure 1).

**Figure 2:**
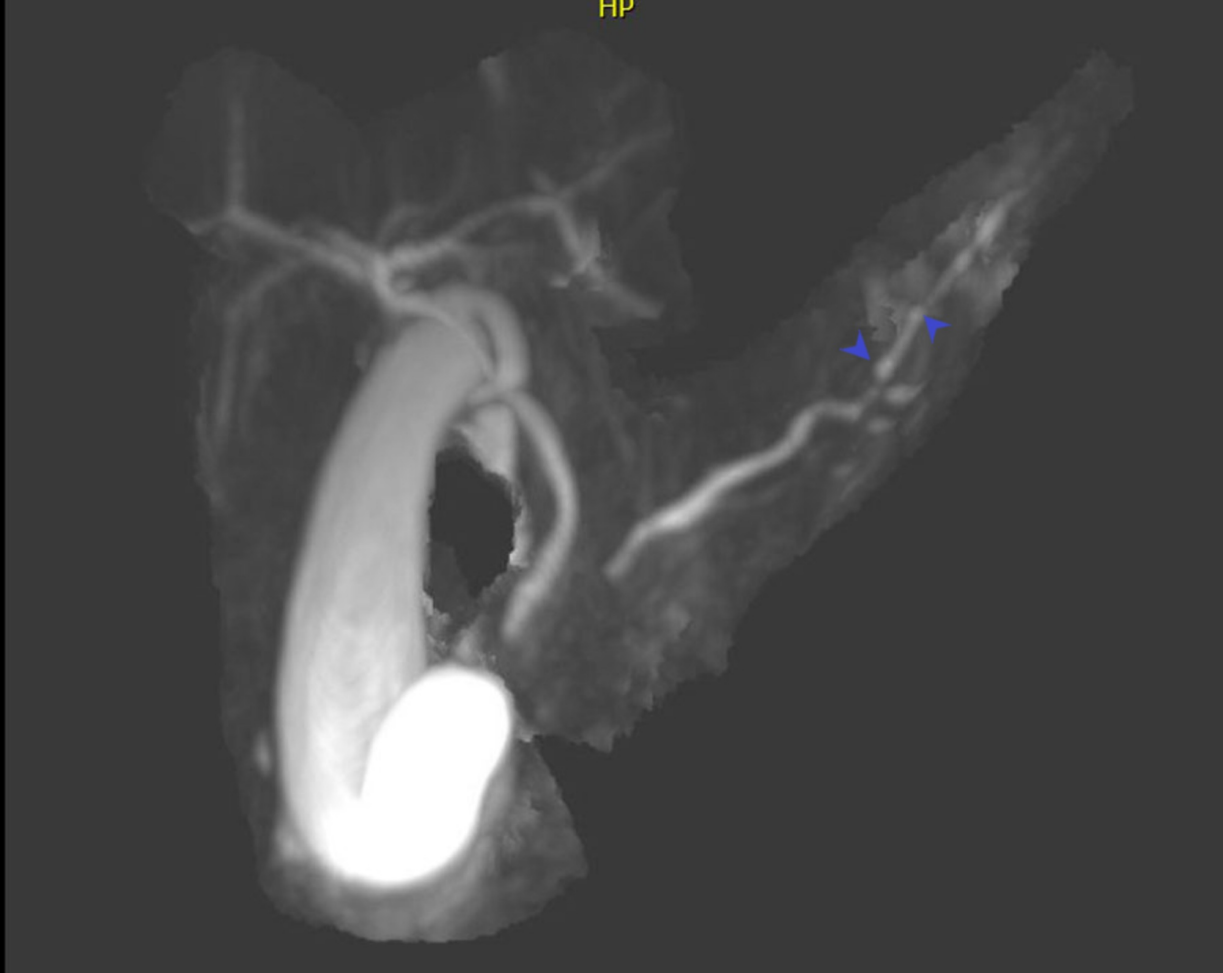
MRI of the pancreas after 3D processing. The image shows edematous pancreas and dilated pancreatic duct with beaded morphology (depicted by blue arrowheads).

Magnetic resonance cholangiopancreatography (MRCP) without intravenous contrast showed marked, diffuse hepatic steatosis, acute-on-chronic interstitial edematous pancreatitis and a dilated main pancreatic duct with beaded morphology in the tail and head (Figure 2).

**Figure 3:**
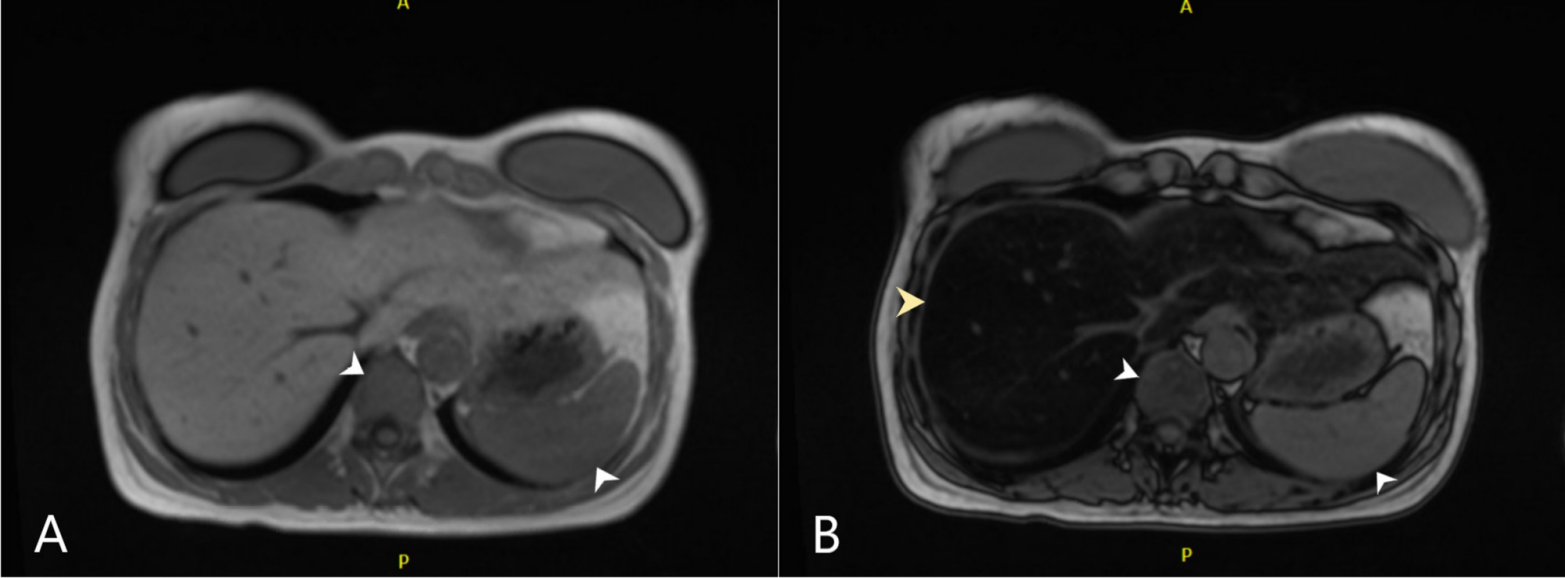
Magnetic resonance cholangiopancreatography T1 W images - in phase (A) and out of phase (B). Panel A shows subtle increase in signal intensity in spleen and bone marrow depicted by arrowheads. Panel B shows shows the same areas as in panel A via white arrowheads. In panel B, the yellow arrowhead depicts the significant loss of signal intensity in the liver owing to fat deposition.

The biliary tree was otherwise unremarkable. There were subtle changes in the signal characteristics in the spleen and marrow compartment suggesting iron deposition (Figure 3A, 3B).

Closer evaluation of the MRCP also showed loss of signal intensity in the pancreatic tail, suggestive of iron deposition. Due to extensive hepatic steatosis, iron deposition in the liver could not be adequately determined. Further lab work showed iron 143 mcg/dL (normal: 35-150 mcg/dL), total iron-binding capacity 166 mcg/dL (normal: 250-450 mcg/dL), transferrin saturation level at 86% (normal: 20%-55%) and ferritin at 692.6 ng/mL (normal: 14.7-205.1 ng/mL). Testing for human homeostatic iron regulator (HFE) gene mutations was done, and the patient tested heterozygous for the C282Y locus, but normal for the H63D and S65C locus. She was diagnosed with acute-on-chronic pancreatitis and hemochromatosis. She was managed conservatively and discharged home with plans for initiation of phlebotomy. The patient's transferrin saturation and ferritin levels normalized over the course of the next few months. There were no further episodes of pancreatitis on follow-up at one year.

## Discussion

Many clinicians believe that hemochromatosis is a rare disorder that is manifested by the clinical findings seen in fully established disease consisting of cirrhosis, diabetes and skin pigmentation (so-called bronze diabetes) [2]. However, these clinical findings are seen only in advanced cases, and many patients present initially with non-specific symptoms. Two types of hemochromatosis have been described: primary (or hereditary) and secondary.

Hereditary hemochromatosis remains the most common identified genetic disorder in Caucasians, with most mutations being in the C282Y, H63D and S65C loci of the HFE gene. However, generally only homozygotes of C282Y locus mutations or compound heterozygotes with C282Y/H63D or C282Y/S65C loci mutations develop overt iron overload. A minor miscellaneous group of hemochromatosis patients has also been identified that do not have any of these mutations [2].

Secondary hemochromatosis, as the name suggests, is due to some underlying cause, such as multiple blood transfusions or excess dietary iron intake. There are certain differences with regards to pattern of iron deposition in the two types of hemochromatosis. In primary hemochromatosis, iron deposition predominantly occurs in the liver and pancreas and less so in the reticuloendothelial system (RES) such as the spleen and bone marrow. In secondary hemochromatosis, iron deposition is seen predominantly in the RES system [3]. MRI is considered an effective imaging modality in assessing iron deposition; however, hepatic steatosis can interfere with determination of iron deposition in the liver unless special MRI technique is used [4]. In our patient, MRI showed iron deposition in the pancreas, spleen and bone marrow but could not adequately assess the liver iron content owing to significant hepatic steatosis.

There have been very few case reports describing hemochromatosis in the African-American population. Labowitz et al. reported a case of hereditary hemochromatosis in an African-American male who was found to be compound C282Y/H63D heterozygote [5]. This is consistent with previous studies suggesting that compound heterozygotes are at an increased risk of developing hemochromatosis. Our patient, however, was neither homozygous for C282Y gene nor a compound heterozygote. Detailed history failed to reveal any cause for iron overload, and it was even more impressive that she developed hemochromatosis in the child-bearing age, given that menstruation slows down development of symptoms of hemochromatosis [6]. The cause of development of hemochromatosis in our patient, therefore, remains unclear.

Chronic pancreatitis is defined as a pathological fibroinflammatory syndrome of the pancreas in individuals with genetic, environmental and/or other risk factors who develop persistent pathological responses to parenchymal injury or stress [7]. Potential causes can include toxic factors (such as alcohol or smoking), metabolic, idiopathic, genetics, autoimmune and obstructive mechanisms [8]. Recurrent attacks of pancreatitis can lead to development of chronic pancreatitis [9]. Recently, a review article by Andersson et al. suggested that hemochromatosis can cause chronic pancreatitis [10]. In our patient, we believe that recurrent attacks of pancreatitis along with hemochromatosis led to the development of chronic pancreatitis.

Our case is unique for several reasons. The patient is an African-American female of child-bearing age. This is important since hereditary hemochromatosis is most common in Caucasians and usually affects females after menopause. The pattern of iron deposition did not seem specific to either primary or secondary hemochromatosis, although liver iron deposition could not be assessed.

From multiple reports, we now know that in patients with hemochromatosis, symptomatic organ involvement typically begins in midlife, often with non-specific symptoms. Liver disease usually predominates but endocrine disorders, cardiac problems and joint disease are also found. Therapeutic phlebotomy is usually effective [11]. Treatment is aimed at reducing total body iron levels and achieving normal ferritin levels. Goals for serum ferritin levels vary between 50 and 150 ng/mL. It is recommended to check hemoglobin levels before each phlebotomy and therapy should be withheld if the hemoglobin level is less than 12.5 gm/dL [12].

## Conclusions

Our patient presented with epigastric pain and investigations revealed acute-on-chronic pancreatitis and evidence of iron overload. She did not have any risk factors for iron overload, such as history of multiple blood transfusions, but tested heterozygous for the C282Y mutation. Pancreatic iron deposition along with alcoholism probably led to the development of acute and eventually chronic pancreatitis. Our case emphasizes that patients with hemochromatosis have a wide range of clinical manifestations, and while liver involvement is the most common, they can present solely with pancreatitis. Pancreatitis with unexplained liver enzyme elevation should prompt evaluation for this rare disorder.
